# P09-02 Risks assessment related to physical activity and sedentary lifestyle profiles among French children and adolescents

**DOI:** 10.1093/eurpub/ckac095.132

**Published:** 2022-08-29

**Authors:** Youssef El Ouadrhiri, Pascale Duché, Anne Vuillemin, Sandrine Carillo, Peggy Pinard, Carine Dubuisson, Irene Margaritis

**Affiliations:** 1 Nutritional Risk Assessment Unit, French Agency for Food, Environmental and Occupational Health & Safety, Maisons-Alfort, France; 2 Université de Toulon, Toulon, France; 3 Université Côte d'Azur, LAMHESS, France

**Keywords:** prevention, risk threshold, behavior

## Abstract

**Introduction:**

Based on Anses's report (2016)[1][1], the French Public Health Policy emphasizes on health status improvement by acting on physical activity and sedentary behaviors. Since 2016, many countries have collected results of large epidemiological studies providing new insights into the effect of physical activity- and mainly sedentary-related behaviors on health. To date, behaviors inducing the highest risks are not identified. In this context, based on the data from the most recent French Food Consumption Survey “(Inca3 2017)”[1][2], Anses aims at characterizing the overweight and obesity risks related to physical fitness regarding the levels of physical activity (PA) and sedentary (SED) daily duration of children and adolescents.

**Methods:**

For 11–17 year-old children, the PA and SED behavior were collected using an adapted Youth Risk Behavior Survey questionnaire. Physical activity and sedentary duration were compared to the thresholds considered as safe (Anses 2016): PA > 60 min/day and SED 20 min/day and SED >4h30/day) were set to disaggregate the population that does not reach these benchmarks, allowing to define nine profiles regarding the associated risks identified and updated in the literature. Profiles were compared according to BMI and age using Pearson chi-square tests. All statistical analysis were performed taking into account the survey complex sampling frame design and the individual weighting.

**Results:**

The preliminary results show nine risk-based profiles of children and adolescents (n = 1285) related to the risk thresholds that can be identified. The most at risk profiles represented almost 50% of the 11-17 years old and were associated with the highest BMI. The highest sedentary (>4h30) profiles were observed in the oldest adolescents. However, among them, regarding the updated literature, those having a high physical activity level were considered as at lower risk.

**Conclusion:**

Finally, definition of profiles based on risk thresholds, >60 min/day and >20 min/day for PA and allows to characterize the children and adolescents the most a risk in order to enhance the effectiveness of public health policy. The risk assessment could be further refined using accelerometer real time measurement of physical activity and sedentary behavior.

